# Assessment of a metabarcoding approach for the characterisation of vector-borne bacteria in canines from Bangkok, Thailand

**DOI:** 10.1186/s13071-019-3651-0

**Published:** 2019-08-08

**Authors:** Lucas G. Huggins, Anson V. Koehler, Dinh Ng-Nguyen, Stephen Wilcox, Bettina Schunack, Tawin Inpankaew, Rebecca J. Traub

**Affiliations:** 1 Faculty of Veterinary and Agricultural Sciences, University of Melbourne, Parkville, VIC 3052 Australia; 2 Faculty of Animal Sciences and Veterinary Medicine, Tay Nguyen University, Buon Ma Thuot, Dak Lak 630000 Vietnam; 3 Walter and Eliza Hall Institute of Medical Research, Parkville, VIC 3052 Australia; 4 Bayer Animal Health GmbH, Leverkusen, Germany; 5 Faculty of Veterinary Medicine, Kasetsart University, Bangkok, 10900 Thailand

**Keywords:** Vector-borne disease, Metabarcoding, Prokaryotic pathogens, Next-generation sequencing, *16S* community profiling, Canines, Tropics, *Mycoplasma*, *Anaplasma*, *Ehrlichia*

## Abstract

**Background:**

Globally, bacterial vector-borne disease (VBD) exerts a large toll on dogs in terms of morbidity and mortality but nowhere is this more pronounced than in the tropics. Tropical environments permit a burgeoning diversity and abundance of ectoparasites some of which can transmit an extensive range of infectious agents, including bacteria, amongst others. Although some of these vector-borne bacteria are responsible for both animal and human diseases in the tropics, there is a scarcity of epidemiological investigation into these pathogens’ prevalence. The situation is further exacerbated by frequent canine co-infection, complicating symptomatology that regular diagnostic techniques may miss or be unable to fully characterise. Such limitations draw attention to the need to develop screening tools capable of detecting a wide range of pathogens from a host simultaneously.

**Results:**

Here, we detail the employment of a next-generation sequencing (NGS) metabarcoding methodology to screen for the spectrum of bacterial VBD that are infecting semi-domesticated dogs across temple communities in Bangkok, Thailand. Our NGS detection protocol was able to find high levels of *Ehrlichia canis*, *Mycoplasma haemocanis* and *Anaplasma platys* infection rates as well as less common pathogens, such as “*Candidatus* Mycoplasma haematoparvum”, *Mycoplasma turicensis* and *Bartonella* spp. We also compared our high-throughput approach to conventional endpoint PCR methods, demonstrating an improved detection ability for some bacterial infections, such as *A. platys* but a reduced ability to detect *Rickettsia*.

**Conclusions:**

Our methodology demonstrated great strength at detecting coinfections of vector-borne bacteria and rare pathogens that are seldom screened for in canines in the tropics, highlighting its advantages over traditional diagnostics to better characterise bacterial pathogens in environments where there is a dearth of research.

**Electronic supplementary material:**

The online version of this article (10.1186/s13071-019-3651-0) contains supplementary material, which is available to authorized users.

## Background

Bacteria transmitted by blood-feeding arthropods, such as ticks and fleas, generate some of the most prevalent and life-threatening illnesses of canines in the tropics [1, 2]. The brown dog tick *Rhipicephalus sanguineus* is of particular importance in such regions, being able to transmit a wide range of different vector-borne diseases (VBDs) including *Ehrlichia canis*, *Anaplasma platys*, and putatively some hemotropic *Mycoplasma* spp., whilst *Ctenocephalides* fleas transmit *Rickettsia felis* and *Bartonella* species [1, 3–6]. One of the most prevalent tick transmitted bacteria contracted by canines in the tropics is *E. canis* the causative agent of canine monocytic ehrlichiosis, which produces a chronic pancytopenia in its later stages that is often fatal [2, 7]. *Anaplasma platys* is another common, tick-borne pathogen of canines that specifically targets platelets, creating a relapsing thrombocytopenia. This pathogen has been observed to reach a prevalence as high as 27% in some regions of tropical northern Australia and when found co-infecting the same host may greatly exacerbate the pathogenesis of other diseases [2, 8, 9]. In fact, the co-infection of multiple vector-borne pathogens in the same canine host is common in the tropics, due to the high diversity of both infectious agents and vectors in such regions, in conjunction with frequently poor access to veterinary care [3, 10].

Assessment and monitoring of canine VBDs is important, not only due to the mortality and morbidity they generate in dogs but also due to the risk they potentially pose to people [1, 11]. Both *Ehrlichia chaffeensis* and *R. felis* can infect canines and are also zoonotic, the former of which is potentially lethal to man and the latter being the aetiological agent of flea-borne spotted fever (FBSF) an emerging zoonosis [8, 12–14]. Furthermore, it is now recognised that the incidence of tick-borne infections in humans, pets and domestic animals is increasing, due to a range of factors, including habitat alteration, greater contact with wildlife and changing population dynamics of the relevant vector [11, 15]. To tackle this, adoption of a One Health approach is of paramount importance, whereby thorough surveillance of VBDs in domestic and wild animal populations is carried out and the reporting of findings disseminated between veterinarians and clinicians to better broadcast and predict the risk of emerging zoonotic threats to man [11, 16].

Detection and diagnosis of bacterial disease has for a long time principally been carried out *via* cultivation on specific growth media, followed by morphological identification with the aid of biochemical and antibiotic testing [17]. Nonetheless, many vector-borne bacteria are unculturable, making serological or molecular techniques preferable [18]. PCR-based diagnosis is particularly useful given that it can provide information on current infection status and can be much more specific than serology which relies on antibodies that often demonstrate interspecific cross-reactivity [19–21]. Such methods do, however, have some limitations including a reliance on the presence of bloodstream circulating pathogens at the time of sampling [22].

With the arrival of next-generation sequencing (NGS) technologies vector-borne bacteria detection and diagnosis has been taken further than what was previously possible, as massive parallelisation of sequencing reactions allows for thorough characterisation of all variants of a sequence of interest [18, 23, 24]. When targeting a barcoding gene, such as the bacterial *16S* small ribosomal RNA subunit locus (*16S* rRNA), a ‘metabarcode’ is created, detailing information on all the bacterial genera and sometimes species present in a sample, depending on the particular *16S* rRNA region targeted and sequence reference library used [23]. NGS-based ‘metabarcoding’ has led to a wealth of research investigating the microbiome of different internal and external environments; however, there is a distinct paucity of research investigating the bacterial blood microbiome in species other than man [24–29]. Moreover, ‘metabarcoding’ is better able to detect novel and rare bacterial species, that family, genus or species-targeting conventional PCR (cPCR) would normally miss [23]. This is of great relevance in regions where there has been little research into vector-borne bacteria of canines, such as in the tropics.

In the present study, we endeavoured to assess for the first time, whether pan-bacterial primers targeting *16S* rRNA could be used to detect vector-borne bacteria of canines with focus on the assay’s ability to detect mixed infections. In addition, we compared this NGS-based method to endpoint cPCR protocols to evaluate differences in both technique’s relative sensitivity and range of species detected. Populations of semi-domesticated Thai dogs were chosen as study subjects, given that canine VBD is known to be highly endemic in these cohorts [30–33].

## Methods

### Sampling and DNA extraction

This study utilised a subset of 100 whole blood samples collected from temple community dogs that make up part of an ongoing project at Kasetsart University, exploring canine and feline VBDs across Thailand. After obtaining informed consent from the relevant monk or caregiver, canine blood samples were collected from 35 Buddhist temple communities. A qualified veterinarian carried out blood sample collection through a cephalic or jugular puncture. This was held in anti-coagulation EDTA tubes and stored at – 20 °C until required. DNA extraction was performed using the E.Z.N.A.® Blood DNA Mini Kit (Omega Biotek Inc., Norcross, GA, USA) from a starting quantity of 250 µl of blood according to the manufacturer’s instructions. The only modification to protocol made was a reduced final DNA elution volume of 100 µl.

### Bacterial *16S* rRNA metabarcoding

The pan-bacterial *16S* rRNA targeting primers 515f Modified (5′-GTG YCA GCM GCC GCG GTA A-3′) from Parada et al. [34] and 806r Modified (5′-GGA CTA CNV GGG TWT CTA AT-3′) from Apprill et al. [35] were chosen, which amplify an approximately 250–300 bp region spanning the *16S* hypervariable 4 (V4) region. This primer pair was selected over others tested, based on its accuracy at identifying VBD present in mock communities that combined between one and five different vector-borne bacterial DNA positive controls. Initially, these primers when tested on canine blood DNA returned many reads identified as canine mitochondrial rRNA sequences, demonstrating cross-reactivity with host DNA (data not shown). To limit this, a degenerate base was removed from both the forward and reverse primers to reduce cross-reactivity, after primer alignment with canine mitochondrial rRNA sequences. The final primers used were Wehi_Adp_515F (5′-GTG YCA GCA GCC GCG GTA A-3′) and Wehi_Adp_806R (5′-GGA CTA CNV GGG TAT CTA AT-3′) with the modified base underlined.

Three separate physical containment areas were utilised for DNA extraction, pre-PCR and post-PCR experiments. All PCRs were prepared in a PCR hood under aseptic conditions following UV sterilisation. Optimal reaction mixtures for amplification were found to be 20 µl comprising 10 µl of OneTaq® 2× Master Mix with Standard Buffer (New England Biolabs, Ipswich, MA, USA) 0.2 μM of both forward and reverse primers, 1 µl of template DNA and 8.2 µl of Ambion Nuclease-Free Water (Life Technologies, Carlsbad, CA, USA). All PCRs were run with positive and no-template negative controls to check for cross-contamination. Field-based DNA extraction negative controls were also run, utilising blood from canines known to be vector-borne bacteria free to ascertain the normal bacterial microbiota of healthy canines from the sampling region and to detect contaminant bacteria from the DNA extraction kits used.

Optimal thermocycling conditions for the selected *16S* rRNA primers were found to be an initial denaturation of 95 °C for 3 min, followed by 35 cycles of 95 °C for 45 s, 56 °C for 60 s and 72 °C for 90 s with a final elongation at 72 °C for 10 min. During PCR optimisation experiments amplicons were run and visualised on a 1.5% agarose gel using a ChemiDoc™ System (Bio-Rad, Hercules, CA, USA).

Deep sequencing of *16S* rRNA amplicon metabarcodes was carried out according to Aubrey et al. [36]. Briefly, the aforementioned first-step PCR was completed with the addition of overhang sequences at the 5′ end of the Wehi_Adp primers. The overhang sequence added to the 5′ end of the forward primer was 5′-GTG ACC TAT GAA CTC AGG AGT C-3′ and to the 5′ end of the reverse primer was 5′-CTG AGA CTT GCA CAT CGC AGC-3′. PCR product was then cleaned using 1× Ampure Beads (Beckman Coulter, Brea, CA, USA). A second PCR step was then carried out introducing eight-base forward and reverse indexing sequences, permitting multiplexing of amplicons onto a single run. Eight forward indexes and 13 reverse indexes were used allowing for multiplexing of 104 bacterial *16S* amplicons, including two no template negative controls and two positive controls (a uniquely identifiable *Rickettsia felis* strain URRWXCal2 from cell culture).

Thermocycling conditions for this second PCR were an initial denaturation of 95 °C for 2 min, followed by 24 cycles of 95 °C for 15 s, 60 °C for 15 s and 72 °C for 30 s with a final elongation at 72 °C for 7 min. Amplicon size distribution was analysed using an Agilent 2200 Tapestation (Agilent, Santa Clara, CA, USA), pooled and then purified using 0.7X Ampure Beads to exclude primer-dimer products [36]. The purified amplicon pool was then quantified using a Qubit 2.0 Fluorometer (Life Technologies, Carlsbad, CA, USA) and run on an Illumina MiSeq (Illumina, San Diego, CA, USA) using 600-cycle v3 chemistry (2 × 300 bp paired-end reads) at the Walter & Eliza Hall Institute Proteomics Facility, Parkville, Australia.

### Bioinformatics analysis

Raw data was demultiplexed using in-house software at the Walter & Eliza Hall Institute and then imported into the QIIME 2 (version 2018.8) environment for bioinformatic processing [37–40]. The cutadapt plugin [41] was used to trim primer, adapter and index sequences from raw reads, followed by inspection for quality, using QIIME2 View. Removal of low quality reads, denoising, dereplicating, filtering of chimeras and merging of forward and reverse reads was then performed using DADA2 [42]. Truncation parameters for DADA2 were decided upon using visual assessment of read quality plots, so that truncation was performed where read quality dropped off (quality score of approximately 35). Next, VSEARCH [43] was used to cluster reads into 97% similarity *de novo* operational taxonomic units (OTUs). The scikit-learn classifier [44] was then used to taxonomically assign these OTU consensus sequences, against the SILVA version 132 reference database, downloaded from docs.qiime2.org. SILVA was preferable to the Greengenes database as it was able to taxonomically assign more OTUs. SILVA-based scikit-learn assignment was corroborated using the BLASTn program in GenBank (NCBI) to taxonomically identify the same OTUs, in some cases this permitted identification to a lower taxonomic level. Unassigned sequences or those only assigned to kingdom and phylum were excluded from the final dataset. Sequences reported from negative control samples were subtracted from the overall dataset and only the results of known, or suspected, vector-borne bacteria were reported. Alpha rarefaction plots were generated, using MAFFT [45] and FastTree 2 [46], to ensure that OTU diversity plateaued and hence a sufficient sequencing depth had been achieved. All NGS data produced in the present study are available from the BioProject database, BioProjectID: PRJNA528154 and SRA data accession numbers SRR8894273 to SRR8894371.

Infections were considered true by NGS, if a sample had a vector-borne bacterial read count of 113 or over. This threshold was determined as the mean reads of four canine DNA samples that were identified as having sequences from the positive controls used within the library preparation, due to occasional index misreading or hybridisation errors during Illumina sequencing [47]. This was supported by assessment of where on the 96-well plate the samples with positive control sequences appeared, which showed no relationship with proximity to positive control location. The average Phred quality score over the adapter and indexing regions for the raw data was 33 which indicates an error rate of between one in 1000 to 10,000, highlighting how occasional sequencing artefacts may have led to index misreading.

### Conventional PCR and Sanger sequencing

To compare the detection ability of our NGS method with traditional molecular techniques all 100 samples were tested for *E. canis*, *A. platys*, *Mycoplasma* spp. and *Rickettsia* Spotted Fever Group (SFG) species by specific endpoint conventional PCR screens from the literature (Table 1).

**Table 1:** Primers used for conventional PCR, real-time PCR and taxonomic cross-validation of NGS results

Taxon targeted	PCR type	Primer pair (5′-3′)	Gene targeted	Product size (bp)	Reference
*Ehrlichia canis*-specific	cPCR	ECA: AACACATGCAAGTCGAACGGA	*16S* rRNA	400	[7]
		HE3: TATAGGTACCGTCATTATCTTCCCTAT			
*Anaplasma platys*-specific	cPCR	PLATYS-F: GATTTTTGTCGTAGCTTGCTATG	*16S* rRNA	678	[50]
		EHR16S-R: TAGCACTCATCGTTTACAGC			
*Mycoplasma* spp.-specific	cPCR	HBT-F: ATACGGCCCATATTCCTACG	*16S* rRNA	600	[48]
		HBT-R: TGCTCCACCACTTGTTCA			
*Rickettsia* Spotted Fever Group	cPCR	ompB-F: CGACGTTAACGGTTTCTCATTCT	Outer membrane protein B (*ompB*)	252	[49]
		ompB-R - ACCGGTTTCTTTGTAGTTTTCGTC			
*Bartonella* spp.-specific	cPCR	prAPT0257: GCCTTCAAGGAGTTGATTTTGTTGTTGCCAAT	Filamenting temperature-sensitive mutant Z (*ftsZ*)	500	[52]
		prAPT0258: ACGACCCATTTCATGCATAACAGAAC			
Filarial worm-specific	cPCR	DIDR-F1: AGTGCGAATTGCAGACGCATTGAG	*5.8S*-ITS2-*28S*	430–660	[53]
		DIDR-R1: AGCGGGTAATCACGACTGAGTTGA			
*Rickettsia* Spotted Fever and Typhus Groups	qPCR	CS-F: TCGCAAATGTTCACGGTACTTT	Citrate synthase (*gltA*)	74	[51]
		CS-R: TCGTGCATTTCTTTCCATTGTG			
		CS-P (Probe): 6-FAM-TGCAATAGAAGAACCGTAGG CTGGATG-BHQ-1			

To confirm vector-borne bacteria identification by NGS, a subset of samples from each taxon were corroborated by Sanger sequencing. This subset of PCR amplicons was purified using the ExoSAP-IT™ PCR Product Cleanup Reagent kit (Thermo Fisher Scientific, Waltham, MA, USA) according to the manufacturer’s protocol. Cleaned amplicons were sent to Macrogen (Seoul, South Korea) for Sanger sequencing.

### Statistical analysis

Analysis of results was conducted in Excel 2016 version 1803 (Microsoft), whilst Kappa statistics to compare concordance of NGS *vs* endpoint cPCR results were calculated in SPSS Statistics 24 (IBM).

## Results

### NGS bioinformatic analysis and characterisation

In total 15,162,431 (median 148,045) raw paired-end reads were obtained for the 104 multiplexed bacterial *16S* amplicons, including two positive and two negative controls. After the DADA2 quality filtering, dereplication, chimera removal and pair-joining step, a total of 7,570,278 (median 73,088) joined sequences (49.9%) were carried forward to the next bioinformatic step. At the OTU clustering stage 723 OTUs were formed of which 94 could not be given any taxonomical assignment and 42 could only be identified to the level of kingdom (39 bacteria; 3 eukaryota). Unassigned OTUs represented 47% of the total filtered reads and were removed from the dataset, many of these sequences were identified as canine mitochondrial rRNA sequences using BLASTn in GenBank. Of the remaining 587 OTUs, 386 were identified down to at least genus level, whilst 42 received a species level classification, using the scikit-learn plug-in. All other OTUs were either assigned to taxonomic levels between kingdom and genus or had top matches with records in the SILVA database that had not originally been classified to the level of species e.g. ‘*Pasteurellaceae* bacterium canine oral taxon’. Positive control DNA sequences were detected at the end of bioinformatic processing. Bacterial sequences found in negative controls were subtracted across all samples in our dataset and only species known to, or suspected to be, vector-borne bacteria were reported. After taxonomic assignment of OTUs eight were from relevant, or suspected, vector-borne bacteria including, *Ehrlichia canis*, *Mycoplasma haemocanis*, “*Candidatus* Mycoplasma haematoparvum”, *Mycoplasma turicensis*, *Anaplasma platys*, *Bartonella* spp., *Rickettsia* spp. and *Wolbachia* spp. A diverse range of other bacterial sequences were also detected *via* our NGS-methodology, but their detection was not the focus of this study, some information regarding these can be found in Additional file 1: Table S1.

From the 100 blood samples tested, our NGS-based detection method found 40 to be positive for *E. canis*, 39 for *Mycoplasma* spp. (34 identified as *M. haemocanis*, three as “*Ca.* M. haematoparvum” and two as *M. turicensis*), 25 for *A. platys* and one for *Bartonella* spp., whilst no samples were found to be positive for *Rickettsia* spp. DNA (Table 2). Other noteworthy taxonomic hits were two samples found positive for *Wolbachia* spp. DNA known to be an endosymbiont of nematodes and arthropods and one sample found positive for *Brucella* spp. DNA. Many commensal or contaminant bacterial species of the skin and environment were also identified from samples (Additional file 1: Table S1).

**Table 2:** Percentage of canine blood samples found positive for a vector-borne bacteria using NGS and conventional PCR screening (*n* = 100 dogs)

Pathogen	NGS % Positive	cPCR % Positive
*Ehrlichia canis*	40	38
*Mycoplasma* spp.	39	40
*Anaplasma platys*	25	12
*Bartonella* spp.	1	1
*Rickettsia* spp.	0	15
*E. canis* + *Mycoplasma* spp.	9	12
*E. canis* + *A. platys*	7	5
*Mycoplasma* spp. + *A. platys*	5	1
*Mycoplasma* spp. + *Bartonella* spp.	1	1
*E. canis* + *Rickettsia* spp.	0	2
*A. platys* + *Rickettsia* spp.	0	1
*Rickettsia* spp. + *Mycoplasma* spp.	0	4
*E. canis* + *A. platys* + *Mycoplasma* spp.	5	2
*E. canis* + *Rickettsia* spp. + *Mycoplasma* spp.	0	2
Total infected dogs	75	74
Total co-infected dogs	27	30

NGS detected a total of 27 bacterial species co-infections, of which 22 comprised two and 5 comprised three canine vector-borne bacteria species. Table 2 shows the number and composition of all bacterial co-infections found. Infections were considered true, if a sample had a vector-borne bacterial read count of 113 or over (see “Methods” for determination of this cut-off).

### Comparison and confirmation of metabarcoding results

Conventional and real-time PCR assays were carried out with which to compare the results of our NGS methodology. Of the 100 canine DNA samples tested, 38 were found positive by an *E. canis*-specific cPCR [7], 40 by a *Mycoplasma* genus-specific cPCR [48], 15 for a *Rickettsia* spp. Spotted Fever Group (SFG) and Transitional Group-specific cPCR [49] and 12 for an *A. platys*-specific cPCR [50]. Combining the results of the separate cPCR screens, 30 dogs were found to be co-infected with vector-borne bacteria; 26 with two bacterial species and 4 with three species (Table 2). In addition, a separate *Rickettsia* genus-specific citrate synthase gene (*gltA*) targeting real-time PCR [51] was conducted to explore differences in PCR detection ability depending on bacterial gene targeted. This real-time PCR assay found all samples to be negative for *Rickettsia* spp., providing 100% agreement with the NGS results for *Rickettsia*.

Table 3 displays the agreement statistics between the NGS and cPCR methodologies. Detection of *Mycoplasma* spp. proved to be the most concordant between the two screening methods with a good level of agreement as defined by the Kappa statistic. Agreement between the two methods when detecting *A. platys* and *E. canis* was not as strong, with both pathogens achieving concordance at a moderate level of agreement, indicating a significant amount of disparity between the results of the two tests for these bacteria. Overall, the two tests demonstrated similar detection capabilities for identifying *E. canis* and *Mycoplasma* spp. as determined by the number of infections found by both tests. However, the NGS method was better at detecting *A. platys* infection, and more varied bacteria such as *Bartonella* spp. or *Wolbachia* spp., in contrast to the cPCR screen which outperformed the NGS method in its ability to detect *Rickettsia* spp. infection.

**Table 3:** Bacterial NGS and cPCR agreement statistics

VBD	cPCR	Bacterial NGS		Total agreement (%)	Kappa^a^ (agreement statistic)	Kappa SE
		POS	NEG			
*E. canis*	POS	26	12	74	0.454 (Moderate)	0.091
	NEG	14	48			
*A. platys*	POS	12	0	87	0.581 (Moderate)	0.098
	NEG	13	75			
*Mycoplasma* spp.	POS	35	5	91	0.812 (Very good)	0.06
	NEG	4	56			

^a^Kappa agreement level: K < 0.2, poor; 0.21–0.40, fair; 0.41–0.60, moderate; 0.61–0.80, good; 0.81–1.00, very good

Cross-validation of NGS results was carried out using endpoint cPCR to amplify larger *16S* rRNA sequences or sequences from other barcoding genes to assess the accuracy of, or improve upon, the level of identification achieved using NGS. In some cases, these were additional to the cPCR assays used to compare detection ability with our NGS method (Table 1). The majority of amplicons produced using an *E. canis*-specific PCR [7] achieved a 100% query cover and identity match with *E. canis* isolate b2-15 (GenBank: KY594915.1) using the GenBank BLASTn tool. A subset of samples identified as *A. platys* by NGS achieved a 100% query cover and identity match with *A. platys* isolate D35 (GenBank: KX792089.2) using an *A. platys*-specific PCR [50]. In addition, the three different *Mycoplasma* species elucidated using NGS were supported by Sanger sequencing, which found a 100% query cover and identity match with *M. haemocanis* isolate F17 (GenBank: KY117659.1) and “*Ca.* M. haematoparvum” (GenBank: KF366443.1) as well as a 99% query cover and 100% identity match with *M. turicensis* isolate F21 (GenBank: KY117663.1).

The sample identified by NGS as having *Bartonella* spp. DNA was successfully cross-validated using a *Bartonella* ftsZ targeting PCR [52] that upon sequencing obtained a 100% query cover and 97% identity match with *Bartonella clarridgeiae* strain 73 (GenBank: FN645454.1).

The two samples that were identified as having *Wolbachia* spp. endosymbiont DNA by NGS were reanalysed using a filarial worm specific PCR [53]. This was done to attempt to elucidate whether the presence of blood-borne *Wolbachia* spp. might represent microfilaremia at the time of sampling, as filarial worms harbour these bacterial endosymbionts [54]. One of the two *Wolbachia* spp.-positive samples amplified a filarial worm specific PCR product using endpoint cPCR and was identified as *Brugia* spp. *via* a BLASTn search with query cover 100% and identity 98–99% to both *Brugia pahangi* (GenBank: EU373655.1) and *Brugia malayi* (GenBank: EU373619.1). One of four randomly tested samples that was *Wolbachia* spp. negative by NGS was amplified using the same filarial worm specific PCR and returned a BLASTn match with *Dirofilaria immitis* clone D2 5.8S (GenBank: JX866681.1; query cover 100%; identity 98%), demonstrating that the presence of *Wolbachia* spp. DNA may highlight filarial infections by some species but not others.

Sanger sequencing of amplicons produced by the *Rickettsia* SFG specific PCR consistently returned BLASTn hits with *Rickettsia asembonensis* (GenBank: LC431491.1; query cover 100%; identity 99%), followed by *Rickettsia felis* clone Ar3 (GenBank: GQ385243.1; query cover 100%; identity 99%), making exact discrimination of the *Rickettsia* spp. involved inconclusive.

## Discussion

To the best of our knowledge this study represents the first use of an Illumina-based NGS detection screen to identify vector-borne bacteria in canine blood. Our method found 40 samples to be positive for *E. canis*, 39 for *Mycoplasma* spp., 25 for *A. platys* and one for *Bartonella* spp. finding an equivalent number of vector-borne bacteria positive dogs when compared to targeted cPCR analysis. Moreover, our method was able to accurately identify bacterial pathogens to species level taxonomic assignment whilst at the same time also identifying rare or unusual pathogens that would not typically be screened for using cPCR assays. Both techniques demonstrated substantial disparity in which bacteria they were able to detect and to what degree. For example, endpoint cPCR screening missed 13 *A. platys* NGS-positive results, whilst our NGS method was unable to detect any of the 15 *Rickettsia* spp. infections detected by endpoint PCR. The two methodologies showed good concordance when detecting *Mycoplasma* spp. DNA from blood. On the other hand, detection of *E. canis* varied greatly between the two techniques, despite both methods finding a similar number of total individuals with *E. canis* infection.

*Anaplasma platys* is an important intracytoplasmic platelet infection of dogs capable of generating thrombocytopenia, fever and lethargy with symptomology being exacerbated during mixed infections with other vector-borne pathogens [8, 12]. Detection of this bacteria was much more sensitive when using our high-throughput approach, compared to the cPCR method by Inokuma et al. [50] as demonstrated by the 13 infections missed by this screen. In the context of *A. platys,* nested conventional PCR screens that first use bacterial generic primers followed by a species specific internal pair have been shown to be more sensitive for the detection of this pathogen in canines, [55] potentially explaining the discrepancy in the current study. The 25 *A. platys* infections found in our subset of Thai dogs was higher than those found in a canine VBD study in the same country which found a prevalence of 4.4% from 181 individuals [30]. Nonetheless, a cPCR as opposed to nested PCR was utilised in this study whilst different sampling locations were also investigated, potentially explaining this difference.

Hemotropic *Mycoplasma* species are ubiquitous pathogens of dogs and other mammals across the globe, able to produce haemolytic anaemia, particularly in immunocompromised hosts [56, 57]. The two most prevalent canine infecting *Mycoplasma* species are *M. haemocanis* and “*Ca.* M. haematoparvum” which were also the most common species identified by our NGS-based protocol in the present study [62, 58]. In the case of this bacterial genus, the results of both detection methods corroborated well, with a high Kappa statistic of 0.812 and a similar number of individuals found infected using both methods. Furthermore, our results are supported by other studies completed in the region which found 19.9% of stray dogs in South Thailand [30] and 12.8% of dogs in northern Cambodia [59] to be infected by a *Mycoplasma* species. The identification of two canines infected with *M. turicensis* was unexpected given that this is typically associated as a pathogen of felines [60]. Nonetheless, this species has been identified in a domestic dog in Brazil [61] and Chile [62], as well as wild animals including Darwin’s foxes [63], lions and ocelots [64], amongst others. Therefore, whether the presence of *M. turicensis* within the Thai dogs sampled in the present study represents sustained infection and transmission in these populations or occasional spill over from wild animals is not possible to ascertain. Nonetheless, it must be acknowledged that molecular-based diagnosis detects the presence of pathogen DNA but does not necessarily provide an indication of current and/or viable infection, despite this often being the case [22].

Although *B. clarridgeiae* has been identified in fleas and cats in Thailand before, to the best of the authors’ knowledge, this is the first report of this species from a dog in the country [65, 66]. *Bartonella clarridgeiae* has been detected in canines previously and shown to cause severe host pathology, such as aortic endocarditis and hepatic disease [67–70]. Furthermore, this species is now a suspected zoonotic pathogen, due to a veterinarian reporting the development of cat-scratch disease (CSD), following the bite of a *B. clarridgeiae* infected cat [70, 65, 66]. Taking this into consideration, the detection of *Bartonella* spp. *via* our NGS method highlights the main benefit of NGS-based techniques to permit the detection of rare and/or unexpected pathogens not typically screened for but potentially able to cause animal and human disease.

The detection of *Wolbachia* spp. endosymbiont sequences *via* NGS in samples from two canines was further explored to assess whether the presence of these sequences could be utilised as a proxy for filarial worm infection [54, 71]. This was supported by a study that demonstrated that *Wolbachia* species phylogeny is largely congruent with that of the filarial worm host [72]. However, a filarial worm-specific cPCR screen [53] only achieved amplification from one of these *Wolbachia-*positive samples, identified as either *B. pahangi* or *B. malayi*, the latter of which is a causative agent of lymphatic filariasis in man [73]. Another sample that was *Wolbachia* sequence negative but was also screened using the filarial PCR assay returned positive amplification for *D. immitis* demonstrating that the presence of *Wolbachia* DNA was an unreliable proxy for infection with filaria. Furthermore, many arthropod vectors also harbour *Wolbachia* endosymbionts and therefore the appearance of this bacteria DNA may represent the incidental presence of *Wolbachia* on the host dog’s skin at the time of sampling, deposited by a dead or passing arthropod [74].

When detecting the important canine pathogen *E. canis*, which generates severe disease in infected individuals [75], the two detection methods assessed differed substantially in which samples they found positive for this bacteria. Twenty-six samples had discordant results; with the NGS methodology finding 14 positive results that were missed by the conventional screen, in comparison to 12 that were missed by NGS. Despite this, both methodologies reported rates of *E. canis* infection higher than the 3.9% rate found previously in Thailand [30]. *Ehrlichia canis* detection can be substantially improved *via* fractionation of blood and targeting of the Buffy Coat layer that acts to concentrate circulating monocytes; the principal cell type infected by this pathogen [12, 75, 76, 77]. Fractionation to test Buffy Coat extracted DNA has been demonstrated to provide good molecular detection of *E. canis* and therefore the absence of this concentration method within our DNA extraction protocol may explain some of the putative missed infections [75]. Furthermore, the *16S* rRNA gene our NGS method targets could be partially responsible for the lower ability to detect infections in some samples. For example, the *E. canis p30* outer membrane protein genes which are present in very high copy numbers per bacterial cell can improve detection ability by as much as 100 times compared to *16S* rRNA based screens [78]. On the other hand, the accuracy of the conventional PCR screen must also be assessed. With this pathogen a nested PCR was not used, therefore, future rectification to use a nested screen [75], alongside replicates [79], would assist in ascertaining the true infection status of such discordant results.

The lack of the NGS-assay’s ability to detect natural *Rickettsia* spp. infection is problematic given that the *R. felis*-complex of rickettsiae are being increasingly detected in dog blood, making the canine host a potential reservoir for this flea-borne zoonosis [14, 59, 80]. In addition, a highly sensitive rickettsial gltA-targeting real-time PCR [51] also found no *Rickettsia* spp. infection. This discrepancy between the cPCR’s ability to detect these infections compared to real-time PCR and NGS is likely due to the target gene used by each technique. To maintain a pan-bacterial range of detection, our NGS method had to use the highly conserved *16S* rRNA gene, whilst the cPCR screen used the rickettsial genus-specific *ompB* gene [81]. It has been established that amplification of outer membrane protein genes is more sensitive than *16S* rRNA targeting, because these genes exist in higher copy numbers per bacterial cell and are therefore easier to detect [49, 82]. PCR-based detection of *Rickettsia* is further exacerbated by typically low quantities of circulating bacteria, especially during chronic, relapsing infections which may have further hindered the ability of our NGS method to detect this genus [83]. Our NGS methodology used positive control DNA from a highly concentrated, cell culture grown *R. felis* strain URRWXCal2, which was detected by NGS. This indicates that the *16S* rRNA primers used by our NGS methodology are capable of amplifying *R. felis* DNA, although potentially not at the concentrations found in natural infections. Future development of our technique may need to consider a supplementary PCR screen using an alternative rickettsial gene target that would improve detection ability and provision species level assignment.

After initial pilot experiments, modifications were made to the 515f [35] and 806r [34] bacterial *16S* rRNA primers to reduce base pair degeneracy and therefore lessen the cross-reactivity potential on host mitochondrial *12S* rRNA sequences. Despite this, as many as 47% of total filtered paired end reads were unable to be taxonomically assigned by the scikit-learn classifier against the SILVA database of which a large proportion represented continued primer cross-reactivity to canine sequences. This is likely due to the sheer abundance of host mitochondrial DNA compared to the relatively small proportion of circulating bacterial DNA, signifying that even with poor primer complementarity to host sequences, the overwhelming amount of these sequences meant bacterial DNA was outcompeted for primer binding. A similar problem was tackled by Gofton et al. [84] within the context of the tick microbiome which is dominated by the endosymbiotic bacterium “*Ca.* Midichloria mitochondri”. These authors used blocking primers to inhibit the amplification of “*Ca.* M. mitochondri” *16S* rRNA sequences during the first round of PCR amplification, allowing better characterisation of the tick microbiome and uncovering of new species that had previously been masked by dominating endosymbiont sequences [84]. Further development of our NGS-based methodology could explore the possibility of a similar approach by preventing mitochondrial sequence amplification and thus improving the detection of low abundance pathogens to augment the assay’s ability to detect vector-borne bacteria in general.

Our deep sequencing method also elucidated many non-pathogenic bacterial OTUs from our canine blood samples with 380 being identified down to genus level (Additional file 1: Table S1). This is likely due to contamination of blood samples during insertion of the collection needle through the skin, hence the prevalence of common skin commensal species such as *Staphylococcus* spp., *Corynebacterium* spp. and *Streptococcus* spp. [85]. Similar findings have been achieved by other researchers working on blood *16S* rRNA metabarcoding, unearthing environmental contaminant species, despite the supposed sterile nature of the blood compartment [24, 27]. The situation is further compounded by the frequent contamination of DNA extraction kits and PCR reagents with *Bradyrhizobium* spp. [86]. This genus, amongst others, was also detected in the present study from negative controls, with such identifications then subtracted from the overall dataset, permitting identification of bacteria arising from the host from those that were commensal or contaminant.

Finally, when comparing our metabarcoding approach with traditional cPCR methods and Sanger sequencing both financial, time and workload considerations must be factored in. For each canine sample to be screened *via* NGS the associated cost was AU$2415 ÷ 104 = AU$23.2 per sample, whilst Sanger sequencing of each positive band typically costs AU$19.9. Given the present results, the total cost of Sanger sequencing of all cPCR positive results would have been 105 × AU$19.9 = AU$2089.5 for detection of the four-principle bacterial groups, i.e. *E. canis*, *A. platys*, *Rickettsia* spp. and *Mycoplasma* spp. This represents a relatively modest price difference, with conventional methods being AU$325.5 cheaper. NGS methods do accrue additional time costs *via* the need for lengthy bioinformatic processing that must be conducted to handle the large datasets they generate. However, the employment of automatic bioinformatic pipelines can make NGS quicker than cPCR when factoring in the additional labour and time required to conduct the multiple cPCR reactions needed to thoroughly characterise pathogen diversity using this technique. Taking all this into consideration, our metabarcoding methodology could be more economical in terms of cost and time spent, particularly if used in areas where canine vector-borne bacteria is even more prevalent than found here, as higher infection levels would require more Sanger sequencing and thus greater per sample expenditure.

## Conclusions

For the first time, we have developed and assessed the use of a *16S* metabarcoding methodology for the simultaneous detection of vector-borne bacteria from canine blood. This assay has proven to be more sensitive than endpoint cPCR and Sanger sequencing for the detection of vector-borne bacteria, such as *A. platys*, better able to characterise rare pathogens and with greater potential to characterise bacterial pathogen species diversity. Despite limitations regarding the detection of *Rickettsia*, this study lays down a crucial framework from which our method can be refined i.e. *via* use of blocking primers to achieve a greater depth of bacterial sequences returned or the use of auxiliary screens for pathogen groups that are difficult to characterise. Our methodology demonstrates great potential as a tool in the armoury of veterinary screens that can be used for surveillance of canine vector-borne bacteria due to its ability to detect rare and novel organisms. This tenet is especially important in the tropics where vector-borne pathogen diversity reaches its peak, but data is limited, whilst also equipping us with a tool that can be used to elucidate and monitor emerging zoonotic threats from these regions [10, 87].

## Additional file

**Additional file 1: Table S1.** Most abundant bacteria not suspected to be vector-borne pathogens. Bacterial species, genera and families detected *via* our NGS methodology that are not suspected pathogens across all samples as a percentage of total reads that passed filtering. *Mycoplasma* spp., *E. canis* and *A. platys* were all more abundant than these commensal or contaminant bacterial groups.

## Data Availability

All NGS data produced in the present study are available from the BioProject database, with BioProjectID: PRJNA528154 and SRA data accession numbers SRR8894273 to SRR8894371. Sanger sequences produced are available from the corresponding author upon reasonable request.
