# Supplementary material for: Assessment of a metabarcoding approach for the characterisation of vector-borne bacteria in canines from Bangkok, Thailand
Source: Parasit Vectors. 2019 Aug 8;12:394. doi: 10.1186/s13071-019-3651-0 (PMC6686542; doi:10.1186/s13071-019-3651-0)
Supplement: Supplementary file 1 — Additional file 1: Table S1. Most abundant bacteria not suspected to be vector-borne pathogens. Bacterial species, genera and families detected via our NGS methodology that are not suspected pathogens across all samples as a percentage of total reads that passed filtering. Mycoplasma spp., E. canis and A. platys were all more abundant than these commensal or contaminant bacterial groups. [file 13071_2019_3651_MOESM1_ESM.docx]

**Additional file 1: Table S1. Most abundant bacteria not suspected to be vector-borne pathogens.** Bacterial species, genera and families detected *via* our NGS methodology that are not suspected pathogens across all samples as a percentage of total reads that passed filtering. *Mycoplasma* spp., *E. canis* and *A. platys* were all more abundant than these commensal or contaminant bacterial groups.

| **Taxonomic Assignment** | **Reads (% of Total)** |
| --- | --- |
| *Stenotrophomonas* spp. | 2.49 |
| Burkholderiaceae | 1.97 |
| *Sediminibacterium* spp*.* | 0.63 |
| *Staphylococcus* spp. | 0.36 |
| Caulobacteraceae | 0.38 |
| *Wolbachia* spp. | 0.37 |
| *Acinetobacter* spp. | 0.27 |
| Enterobacteriaceae | 0.23 |
| Geminicoccaceae | 0.20 |
| *Pseudomonas* spp. | 0.19 |
| *Enhydrobacter* spp. | 0.19 |
| *Corynebacterium* spp. | 0.17 |
| *Bradyrhizobium* spp. | 0.17 |
| *Sphingomonas* spp. | 0.15 |
| *Pasteurellaceae* spp. | 0.14 |
| *Anaerobacillus* spp. | 0.11 |
| *Streptococcus* spp. | 0.10 |
